# Treatment discontinuation among users of GLP-1 receptor agonists and SGLT2 inhibitors in a national population of individuals with type 2 diabetes

**DOI:** 10.1007/s00125-025-06439-x

**Published:** 2025-05-02

**Authors:** Carl-Emil Lim, Björn Pasternak, Björn Eliasson, Peter Ueda

**Affiliations:** 1 Clinical Epidemiology Division, Department of Medicine, Solna, Karolinska Institutet, Stockholm, Sweden; 2 Department of Epidemiology Research, Statens Serum Institut, Copenhagen, Denmark; 3 Department of Medicine, Sahlgrenska University Hospital, Gothenburg, Sweden

**Keywords:** Adherence, Discontinuation, Drug switching, GLP-1 receptor agonists, Persistence, Proportion of patients covered, Reinitiation, SGLT2 inhibitors, Type 2 diabetes

## Abstract

**Aims/hypothesis:**

Our aim was to assess treatment discontinuation, reinitiation and switching between drugs within the same drug class for glucagon-like peptide-1 (GLP-1) receptor agonists and sodium-glucose cotransporter 2 (SGLT2) inhibitors in individuals with type 2 diabetes.

**Methods:**

We used data from nationwide registers in Sweden to perform separate analyses for all patients with type 2 diabetes who filled a first prescription of a GLP-1 receptor agonist or an SGLT2 inhibitor between 2017 and 2021. Patients were considered to be on treatment for the period during which prescriptions were refilled before the estimated end date of the most recent prescription, including a 90-day grace period, i.e. the time allowed between and after prescriptions before treatment is considered as discontinued. We used the Aalen–Johansen estimator to estimate cumulative incidences of discontinuation and reinitiation, and Fine–Gray sub-distribution hazard models to assess the association of clinical variables with the risk of discontinuation.

**Results:**

Among 73,895 new users of GLP-1 receptor agonists, the cumulative incidence of treatment discontinuation was 23.6% at 1 year and 38.5% at 3 years. Among patients who discontinued, the cumulative incidence of treatment reinitiation was 41.1% at 1 year and 57.4% at 3 years after discontinuation. Among 113,207 new users of SGLT2 inhibitors, the cumulative incidence of treatment discontinuation was 27.9% at 1 year and 45.9% at 3 years, with a cumulative incidence of reinitiation of 40.4% at 1 year and 55.7% at 3 years after discontinuation. When varying the grace period between 60 days and 365 days, treatment discontinuation rates at 3 years ranged from 23.3% to 43.6% among GLP-1 receptor agonist users and from 28.8% to 50.6% among SGLT2 inhibitor users. The proportion of patients who had ongoing treatment, regardless of previous discontinuation episodes, ranged between approximately 70% and 80% for both drugs during a 1–5 year period after treatment initiation across analyses using various grace periods. In terms of switching, 22.9% of the GLP-1 receptor agonist users and 2.1% of the SGLT2 inhibitor users switched between drugs within the same drug class. Patient characteristics associated with treatment discontinuation were similar for GLP-1 receptor agonists and SGLT2 inhibitors, although the association between higher BMI and a lower likelihood of treatment discontinuation was stronger for GLP-1 receptor agonists.

**Conclusions/interpretation:**

Approximately half of type 2 diabetes patients who had started using GLP-1 receptor agonists or SGLT2 inhibitors had discontinued treatment within 5 years of follow-up. However, more than half of those who discontinued treatment subsequently reinitiated treatment, such that the proportion with ongoing treatment was approximately 70–80% for both drugs during a 1–5 year period after treatment initiation. This suggests that the proportion of patients with long-term use of the medications is larger than indicated by analyses focusing on treatment discontinuation. Patient characteristics associated with treatment discontinuation were similar for GLP-1 receptor agonists and SGLT2 inhibitors.

**Graphical Abstract:**

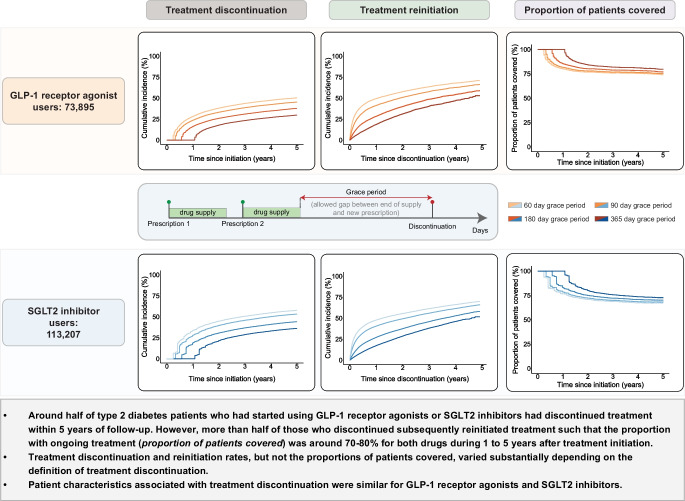

**Supplementary Information:**

The online version contains peer-reviewed but unedited supplementary material available at 10.1007/s00125-025-06439-x.


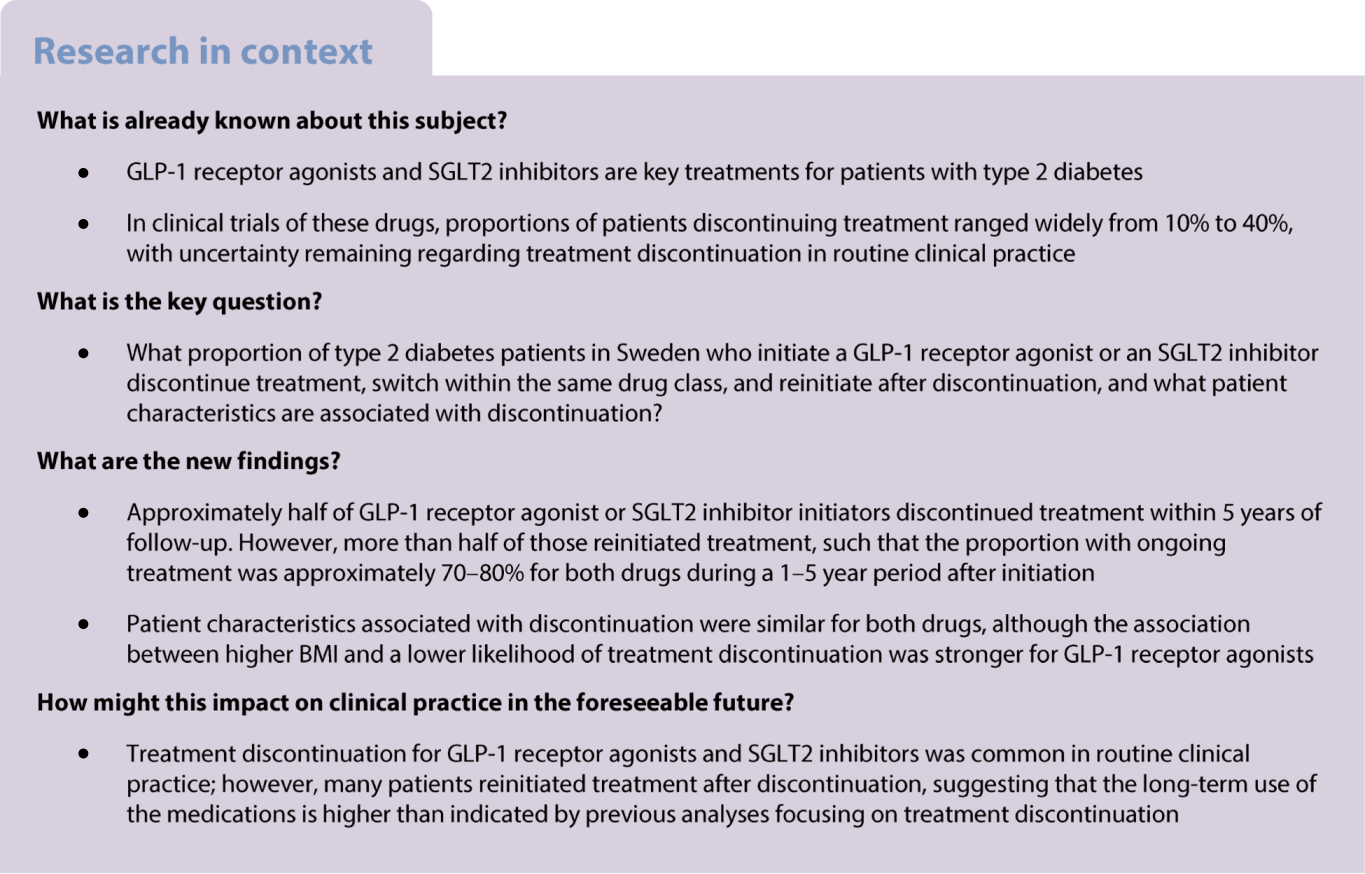


## Introduction

Glucagon-like peptide-1 (GLP-1) receptor agonists and sodium-glucose cotransporter 2 inhibitors (SGLT2) are key treatments for type 2 diabetes that improve cardiovascular and renal outcomes [1–3]. International guidelines now recommend use of either drug for most patients with type 2 diabetes [4–6]. Although continued treatment is necessary for patients to benefit, uncertainty remains regarding rates of treatment discontinuation in routine clinical practice.

In clinical trials of GLP-1 receptor agonists, treatment discontinuation has varied between studies and between individual drugs [7–13]. For example, the total discontinuation rates were 27% in the SUSTAIN 6 trial of semaglutide (mean follow-up 39.8 months) and 42% in the REWIND trial of dulaglutide (median follow-up 65 months). Additionally, while the total discontinuation rates were not reported, the proportion of patients receiving active treatment who discontinued due to adverse events was 10% in the LEADER trial of liraglutide (median follow-up 46 months) and 12% in the PIONEER 6 trial of oral semaglutide (median follow-up 15.9 months). The reported discontinuation rates were not substantially higher compared with those receiving placebo.

In clinical trials of SGLT2 inhibitors in type 2 diabetes, discontinuation rates among those receiving active treatment have been less variable, ranging from 21% during a median follow-up time of 50 months in the DECLARE-TIMI 58 trial of dapagliflozin to 29% during a mean follow-up time of 43 months in the CANVAS trial of canagliflozin [14–18].

Clinical trials are performed in highly controlled settings, with patient monitoring and encouragement to adhere to the assigned treatment. Rates of treatment discontinuation are therefore expected to be higher in routine clinical practice. On the other hand, in contrast to those enrolled in clinical trials, patients seen in routine clinical practice are allowed to switch between drugs within the same drug class, which could lower rates of treatment discontinuation.

In this study, we used nationwide registers in Sweden to assess treatment discontinuation, reinitiation and switching for GLP-1 receptor agonists and SGLT2 inhibitors in patients with type 2 diabetes.

## Methods

### Data sources

Data on study drugs and co-medications was obtained from the Swedish National Prescription Drug Register [19], which holds individual-level information on all drug prescriptions filled at all pharmacies in Sweden since July 2005. The register includes information on the anatomical therapeutic chemical code of the dispensed drug, the amount of drug dispensed, dosage form (pill or injection), and the date when the prescription was filled.

We obtained data on diabetes type, HbA_1c_ level, BP, eGFR and smoking status from the Swedish National Diabetes Register. For this nationwide register, data are collected by physicians and nurses during visits to outpatient and primary care clinics by patients with type 1 or type 2 diabetes in Sweden. During the period for patient inclusion in our study (2017–2021), 85–96.5% of all patients receiving drugs for diabetes in Sweden (as registered in the National Prescription Drug Register) were included in the register [20–24].

We obtained data on procedure codes and diagnoses according to the International Classification of Diseases, tenth revision (ICD-10; https://icd.who.int/browse10/2019/en), from the National Patient Register [25]. These are assigned by physicians during hospital admissions and outpatient specialist care visits in Sweden.

Individual-level data on age, sex, vital status, place of birth, educational level and income were obtained from the Total Population Register, which is maintained by Statistics Sweden. The Total Population Register has been described in detail previously [26]. Data on race and ethnicity were not available.

Individual consent is not required for inclusion of patients in national health registries according to Swedish law. Conduct of the study was approved by the Regional Ethics Committee in Stockholm, Sweden.

### Study population

We included all patients registered as having type 2 diabetes in the National Diabetes Register who filled their first prescription for a GLP-1 receptor agonist, and, in a separate cohort, all patients registered as having type 2 diabetes in the National Diabetes Register who filled their first prescription for an SGLT2 inhibitor, between 1 January 2017 and 31 December 2021 (ensuring a minimum of 1-year follow-up until the end of the study period on 31 December 2022). The date of filling the first prescription was defined as the index date. Patients were included in both cohorts if they had filled a first prescription for both drug classes.

### Treatment discontinuation and reinitiation

The primary outcomes were treatment discontinuation and treatment reinitiation. Patients were considered as receiving the study drug class for the period during which prescriptions for the same drug class were refilled before the estimated end date of the most recent prescription, including a grace period of 90 days to account for irregular drug use. Anatomical therapeutic chemical codes and estimated days of supply are provided in electronic supplementary material [ESM] Table 1. If a patient refilled a prescription before the end date of supply of the preceding prescription, resulting in an overlap in medication supply, we applied a stockpiling approach, i.e. it was assumed that the patient used up all medication from the previous prescription before using the medication from the refill prescription.

Treatment discontinuation was defined as no refill within 90 days after the estimated end date of the most recent prescription. For patients who discontinued treatment during the study period, we used the day of treatment discontinuation (defined as the estimated end date of the most recent prescription plus the grace period of 90 days) as the start of follow-up to assess treatment reinitiation, defined as the first new prescription of the drug class that had previously been discontinued.

### Treatment trajectories and switching  

For patients who started treatment between 1 January 2017 and 31 December 2019, we described treatment trajectories during a 3 year period after treatment initiation, including up to five of the following events: (1) first switch within the same drug class, (2) treatment discontinuation, (3) reinitiation, (4) death or emigration, and (5) continued treatment. Patients who filled a prescription for another drug within the same drug class were considered as having switched their medication. The GLP-1 receptor agonists were categorised as semaglutide, liraglutide and ‘other GLP-1 receptor agonists’. The SGLT2 inhibitors were categorised as empagliflozin, dapagliflozin and ‘other SGLT2 inhibitors’. Switches within the categories of other GLP-1 receptor agonists and other SGLT2 inhibitors were considered as switches but were not further categorised due to the low number of such switches.

### Treatment adherence

Treatment adherence was assessed by calculating the proportion of days covered, defined as the number of days covered by the study drug (excluding the grace period) at 1 year after the date of filling the first prescription, divided by 365 days, for all patients who were alive at 1 year after drug initiation.

### Statistical analysis

All analyses were performed separately in each cohort. Patients were followed from the index date until first occurrence of treatment discontinuation, emigration, death or the end of the study period (31 December 2022). For the analyses of treatment reinitiation, patients were followed from the day of treatment discontinuation until first occurrence of treatment reinitiation, emigration, death or the end of the study period. We described the cumulative incidence of treatment discontinuation in all patients and treatment reinitiation among those who discontinued treatment using the Aalen–Johansen estimator. We used Fine–Gray sub-distribution hazard model analyses adjusted for sex and age to assess the association of sociodemographic variables, comorbidities and co-medications (defined in ESM Table 2) with the risk of treatment discontinuation. The Aalen–Johansen estimator and the Fine–Gray sub-distribution hazard models were used to account for the competing risk of death. We performed additional analyses of treatment discontinuation and reinitiation in which we used grace periods of 60, 180 and 365 days.

We performed subgroup analyses of treatment discontinuation in patients according to the presence of atherosclerotic cardiovascular disease (ASCVD), chronic kidney disease or heart failure (ESM Table 3), BMI category (<25, ≥25 to <30, ≥30 to <35 and ≥35 kg/m^2^) and calendar year.

Given the proportions of missing data for eGFR, BMI and other variables (ESM Table 2), we used multiple imputations (Markov chain Monte Carlo method) to handle missing data. We imputed ten datasets by predictive mean matching for continuous variables, logistic regression for dichotomous variables, and multinomial logistic regression for polytomous variables. All analyses that included missing variables were run across all imputed datasets and then pooled in accordance with Rubin’s rules [27].

We assessed the proportion of patients who adhered to treatment according to proportion thresholds of ≥80, ≥90 and 100% of days covered [28–30].

As discontinuation and reinitiation rates varied significantly depending on the length of the grace period used to define treatment discontinuation, and as many patients reinitiated treatment after discontinuation, we performed a post hoc analysis to assess the proportion of patients covered by treatment [31]. The proportion of patients covered was calculated by dividing the number of patients with ongoing treatment by the total number of patients who were alive and who had not emigrated at each given time point. Ongoing treatment was defined with the exposure definition used in the main analyses, including a grace period of 90 days, with patients being allowed to discontinue and reinitiate treatment multiple times. We used the proportion of patients covered as it is less affected by the length of the grace period compared with time-to-event analyses, and takes not only discontinuation but also reinitiation of treatment into account [31]. In additional post hoc analyses, we calculated the proportion of patients covered using grace periods of 30, 60, 180 and 365 days.

All analyses were performed using R version 4.3.2 (R Foundation for Statistical Computing, Vienna, Austria).

## Results

### Study population

We identified 73,895 new users of GLP-1 receptor agonists and 113,207 new users of SGLT2 inhibitors (Table 1). Among GLP-1 receptor agonist users, the mean age (± SD) was 62.9 ± 12.2 years, 58.8% were men, 67.1% were obese (BMI ≥30 kg/m^2^) and 23.0% had a history of ASCVD. Among SGLT2 inhibitors users, the mean age (± SD) was 65.1 ± 11.5 years, 64.8% were men, 10.7% had a history of heart failure, 15.3% had a history of chronic kidney disease, and 29.2% had a history of ASCVD.

**Table 1:** Characteristics of new users of SGLT2 inhibitors and GLP-1 receptor agonists

	SGLT2 inhibitors (*N* = 113,207)	GLP-1 receptor agonists (*N* = 73,895)
Male	73,386 (64.8)	43,486 (58.8)
Age (years)		
<40	2544 (2.2)	2946 (4.0)
40–49	8376 (7.4)	7198 (9.7)
50–59	22,990 (20.3)	17,040 (23.1)
60–69	35,385 (31.3)	22,340 (30.2)
70–79	34,294 (30.3)	19,636 (26.6)
≥80	9618 (8.5)	4735 (6.4)
Place of birth		
Nordic countries^a^	87,820 (77.6)	58,628 (79.3)
Outside Europe	15,716 (13.9)	9343 (12.6)
Rest of Europe	9671 (8.5)	5924 (8.0)
Education		
Primary school and high school	88,388 (78.1)	57,263 (77.5)
Vocational or short-term tertiary education	12,271 (10.8)	8151 (11.0)
Medium- or long-term tertiary education	12,548 (11.1)	8481 (11.5)
Income		
Low income	28,338 (25.0)	18,505 (25.0)
Middle income	56,546 (49.9)	36,903 (49.9)
High income	28,323 (25.0)	18,487 (25.0)
Living with partner	58,433 (51.6)	36,113 (48.9)
Medical history		
ASCVD	33,018 (29.2)	16,973 (23.0)
Ischaemic heart disease or coronary revascularisation	26,617 (23.5)	12,875 (17.4)
Ischaemic stroke	6173 (5.5)	3494 (4.7)
Arterial disease (including amputation)	5878 (5.2)	3751 (5.1)
Atrial fibrillation	13,754 (12.1)	7418 (10.0)
Heart failure	12,166 (10.7)	6140 (8.3)
Chronic kidney disease	17,305 (15.3)	16,058 (21.7)
Liver disease	3036 (2.7)	2250 (3.0)
Pancreatitis	1621 (1.4)	934 (1.3)
Diabetic eye complications	17,349 (15.3)	11,914 (16.1)
Other diabetic complications	17,088 (15.1)	12,573 (17.0)
Psychiatric disorder	17,510 (15.5)	13,068 (17.7)
Mental and behavioural disorders due to psychoactive substance use	6719 (5.9)	4434 (6.0)
Prescription drugs in previous year		
SGLT2 inhibitors	–	19,701 (26.7)
GLP-1 receptor agonists	19,292 (17.0)	–
Insulin	34,192 (30.2)	28,887 (39.1)
Other non-insulin diabetes drugs^b^	103,933 (91.8)	66,667 (90.2)
Platelet inhibitors	10,135 (9.0)	4539 (6.1)
Statins	84,101 (74.3)	52,055 (70.4)
ACE inhibitors/angiotensin II receptor blockers	81,000 (71.6)	51,432 (69.6)
Calcium antagonists	41,717 (36.9)	27,207 (36.8)
β-blockers	53,099 (46.9)	31,555 (42.7)
Diuretics	30,280 (26.7)	20,074 (27.2)
Time since first diabetes drug (years)		
<1	9865 (8.7)	6237 (8.4)
1–9	59,542 (52.6)	38,420 (52.0)
≥10 years	43,800 (38.7)	29,238 (39.6)
Number of diabetes drugs^c^		
0	8021 (7.1)	6480 (8.8)
1 or 2	100,091 (88.4)	59,503 (80.5)
≥3	5095 (4.5)	7912 (10.7)
NDR variables		
BP		
Normotension	65,048 (57.5)	43,206 (58.5)
Stage 1 hypertension	40,759 (36.0)	26,350 (35.7)
Stage 2 hypertension	7400 (6.5)	4339 (5.9)
HbA_1c_		
≤48 mmol/l (≤6.5%)	11,891 (10.5)	6959 (9.4)
49–52 mmol/l (6.6–6.9%)	10,893 (9.6)	5875 (8.0)
53–63 mmol/l (7.0–7.9%)	38,555 (34.1)	22,942 (31.0)
64–74 mmol/l (8.0–8.9%)	26,365 (23.3)	18,563 (25.1)
≥75 mmol/l (≥9.0%)	25,503 (22.5)	19,556 (26.5)
BMI		
Normal weight	12,856 (11.4)	4371 (5.9)
Overweight	39,925 (35.3)	19,946 (27.0)
Obese class I	36,159 (31.9)	25,486 (34.5)
Obese class II/III	24,267 (21.4)	24,092 (32.6)
eGFR (ml/min per 1.73 m^2^)		
≥90	49,629 (43.8)	31,942 (43.2)
60–89	50,281 (44.4)	28,133 (38.1)
30–59	12,969 (11.5)	12,995 (17.6)
15–29	313 (0.3)	771 (1.0)
<15	15 (0.0)	54 (0.1)
Smoking	15,673 (13.8)	10,191 (13.8)

Values are *n* (%). Detailed definitions of characteristics are provided in ESM Table 2

^a^The Nordic countries are Sweden, Norway, Denmark and Finland

^b^Metformin, sulfonylureas, dipeptidyl peptidase 4 (DPP4) inhibitors, thiazolidinediones, glinides, acarbose

^c^Not including insulin in both cohorts, SGLT2 inhibitors in the SGLT2 inhibitor cohort, and GLP-1 receptor agonists in the GLP-1 receptor agonist cohort

ACE, angiotensin-converting-enzyme; NDR, National Diabetes Register

### Treatment discontinuation

**Fig. 1:**
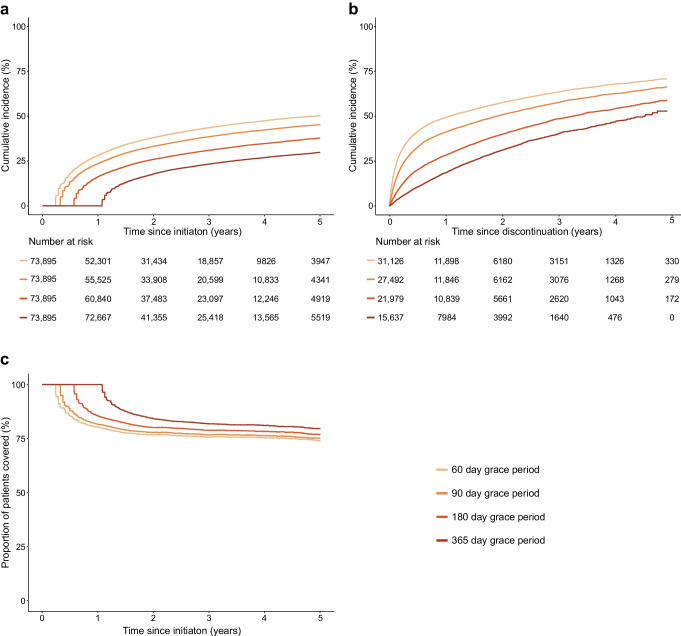
Treatment discontinuation (**a**), treatment reinitiation (**b**) and the proportion of patients covered by treatment (**c**) across various lengths of grace period used to define treatment discontinuation among GLP-1 receptor agonist users

**Fig. 2:**
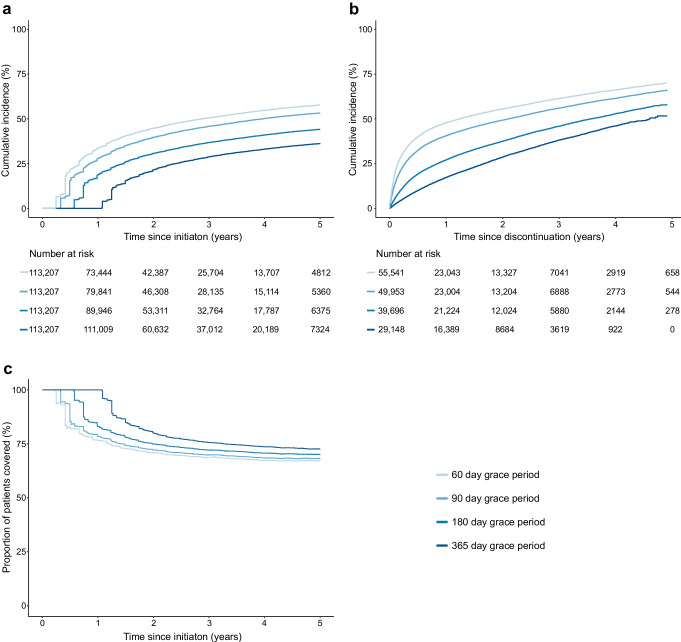
Treatment discontinuation (**a**), treatment reinitiation (**b**) and the proportion of patients covered by treatment (**c**) across various lengths of grace period used to define treatment discontinuation among SGLT2 inhibitor users

The cumulative incidences of treatment discontinuation and reinitiation among GLP-1 receptor agonist users and SGLT2 inhibitor users using various length of grace periods are shown in Figs 1 and 2, respectively.

For GLP-1 receptor agonist users, the cumulative incidence of treatment discontinuation was 23.6% (95% CI 23.2, 23.9) at 1 year and 38.5% (95% CI 38.2, 38.9) at 3 years, when using a grace period of 90 days. Discontinuation rates were substantially reduced to 16.2% (95% CI 16.0, 16.5) at 1 year and 31.0% (95% CI 30.6, 31.4) at 3 years when using a grace period of 180 days, and to 23.3% (95% CI 23.0, 23.7) at 3 years when using a grace period of 365 days. Conversely, discontinuation rates were increased to 28.1% (95% CI 27.7, 28.4) at 1 year and 43.6% (95% CI 43.2, 44.0) at 3 years when using a grace period of 60 days (ESM Table 4).

For SGLT2 inhibitor users, the cumulative incidence of treatment discontinuation was 27.9% (95% CI 27.6, 28.1) at 1 year and 45.9% (95% CI 45.5, 46.2) at 3 years, when using a grace period of 90 days. Discontinuation rates were substantially reduced to 18.8% (95% CI 18.6, 19.0) at 1 year and 36.8% (95% CI 36.5, 37.1) at 3 years when using a grace period of 180 days, and to 28.8% (95% CI 28.5, 29.1) at 3 years when using a grace period of 365 days. Conversely, discontinuation rates were increased to 33.7% (95% CI 33.4, 34.0) at 1 year and 50.6% (95% CI 50.3, 50.9) at 3 years when using a grace period of 60 days (ESM Table 4).

**Fig. 3:**
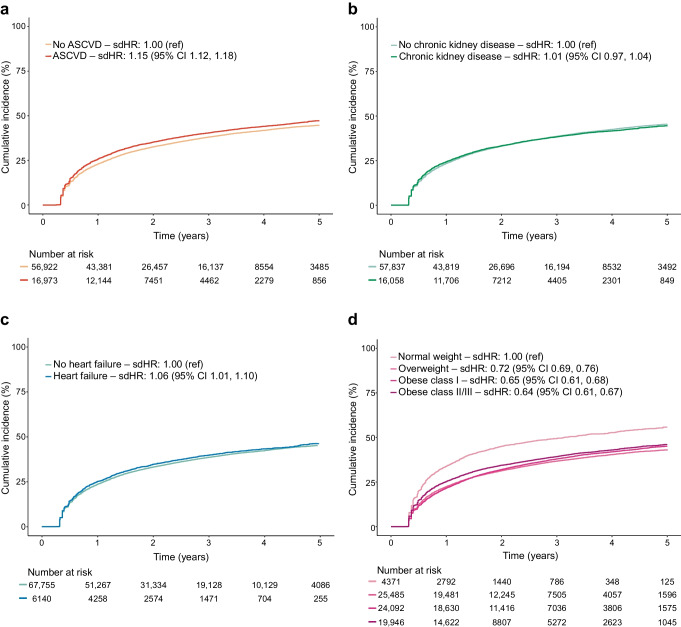
Treatment discontinuation across subgroups of ASCVD (**a**), chronic kidney disease (**b**), heart failure (**c**) and BMI (**d**) among GLP-1 receptor agonist users. Cut-offs for BMI classes are given in ESM Table 2. sdHR, sub-distributional HR

**Fig. 4:**
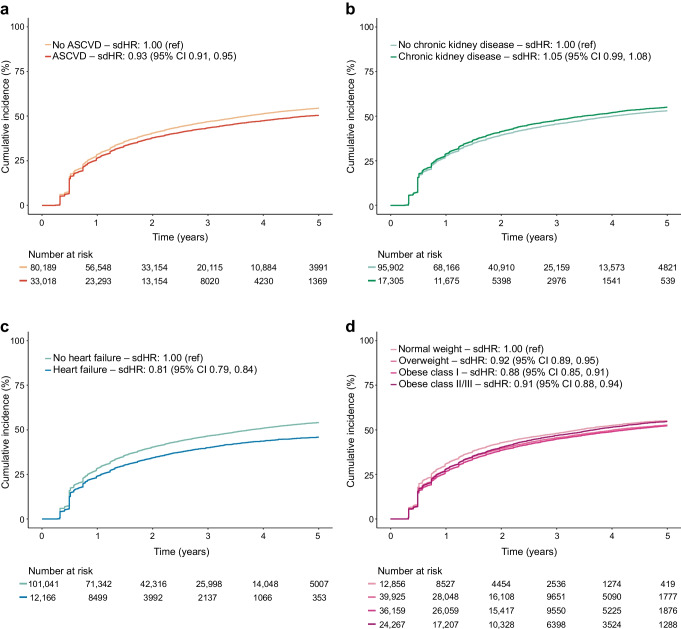
Treatment discontinuation across subgroups of ASCVD (**a**), chronic kidney disease (**b**), heart failure (**c**) and BMI (**d**) among SGLT2 inhibitor users. Cut-offs for BMI classes are given in ESM Table 2. sdHR, sub-distributional HR

Figure 3 shows that, while there were no or small differences in discontinuation rates between subgroups according to status of ASCVD, chronic kidney disease and heart failure among users of GLP-1 receptor agonists, discontinuation rates were higher among those with normal weight vs other weight categories. For example, the HR for treatment discontinuation was 0.65 (95% CI 0.61, 0.68) for obesity class II/III vs normal weight (Table 2). Figure 4 shows that discontinuation rates for SGLT2 inhibitors differed little between subgroups of patients by status of ASCVD, chronic kidney disease and heart failure, although some of the differences were statistically significant. The largest difference was seen among patients with heart failure vs those without (HR for discontinuation 0.81, 95% CI 0.79, 0.84) (Table 2).

**Table 2:** Sex- and age adjusted sub-distributional HRs for discontinuation of treatment in new users of GLP-1 receptor agonists and SGLT2 inhibitors

Characteristics	GLP-1 receptor agonists	SGLT2 inhibitors
Male	0.95 (0.93, 0.98)	0.84 (0.82, 0.85)
Age (years)		
<40	Reference	Reference
40–49	0.73 (0.69, 0.78)	0.74 (0.70, 0.78)
50–59	0.61 (0.58, 0.64)	0.63 (0.60, 0.67)
60–69	0.57 (0.54, 0.60)	0.60 (0.58, 0.64)
70–79	0.61 (0.58, 0.64)	0.65 (0.62, 0.68)
≥80	0.77 (0.72, 0.82)	0.70 (0.66, 0.74)
Place of birth		
Nordic countries^a^	Reference	Reference
Outside Europe	1.67 (1.62, 1.73)	1.17 (1.14, 1.20)
Rest of Europe	1.33 (1.27, 1.38)	1.16 (1.12, 1.19)
Education		
Primary school and high school	Reference	Reference
Vocational or short-term tertiary education	0.97 (0.94, 1.01)	0.95 (0.92, 0.97)
Medium- or long-term tertiary education	0.92 (0.89, 0.96)	0.92 (0.89, 0.94)
Income		
Low income	Reference	Reference
Middle income	0.83 (0.81, 0.86)	0.90 (0.88, 0.92)
High income	0.70 (0.68, 0.72)	0.79 (0.77, 0.81)
Living with partner	0.92 (0.90, 0.94)	0.95 (0.93, 0.96)
Medical history		
ASCVD	1.15 (1.12, 1.18)	0.93 (0.91, 0.95)
Ischaemic heart disease and coronary revascularisation	1.14 (1.10, 1.17)	0.92 (0.90, 0.94)
Ischaemic stroke	1.10 (1.04, 1.16)	0.92 (0.88, 0.95)
Arterial disease (including amputation)	1.14 (1.09, 1.21)	1.03 (0.99, 1.07)
Atrial fibrillation	0.98 (0.95, 1.03)	0.83 (0.81, 0.86)
Heart failure	1.06 (1.01, 1.10)	0.81 (0.79, 0.84)
Chronic kidney disease	1.01 (0.97, 1.04)	1.05 (0.99, 1.08)
Liver disease	1.18 (1.11, 1.26)	1.07 (1.01, 1.12)
Pancreatitis	1.24 (1.13, 1.37)	1.04 (0.97, 1.12)
Diabetic eye complications	1.16 (1.12, 1.19)	1.00 (0.98, 1.03)
Other diabetic complications	1.19 (1.16, 1.23)	1.01 (0.98, 1.03)
Psychiatric disorder	1.14 (1.11, 1.18)	1.07 (1.04, 1.09)
Mental and behavioural disorders due to psychoactive substance use	1.17 (1.12, 1.23)	1.08 (1.04, 1.12)
Prescription drugs in previous year		
Insulin	1.17 (1.14, 1.20)	1.04 (1.02, 1.06)
Cardiovascular drugs^b^	0.79 (0.76, 0.82)	0.73 (0.71, 0.75)
Diuretics	0.96 (0.94, 0.99)	0.89 (0.87, 0.91)
Time since first diabetes drug (years)		
<1	Reference	Reference
1–9	0.89 (0.86, 0.93)	1.06 (1.02, 1.09)
≥10	0.97 (0.93, 1.02)	1.05 (1.02, 1.09)
Number of diabetes drugs^c^		
0	Reference	Reference
1 or 2	0.76 (0.73, 0.79)	0.83 (0.81, 0.86)
≥3	0.65 (0.62, 0.69)	0.71 (0.68, 0.75)
NDR variables		
BP		
Normotension	Reference	Reference
Stage 1 hypertension	1.01 (0.98, 1.04)	1.04 (1.02, 1.06)
Stage 2 hypertension	1.12 (1.07, 1.18)	1.06 (1.02, 1.11)
HbA_1c_		
≤48 mmol/l (≤6.5%)	Reference	Reference
49–52 mmol/l (6.6–6.9%)	0.90 (0.84, 0.95)	1.07 (1.03, 1.12)
53–63 mmol/l (7.0–7.9%)	0.90 (0.86, 0.94)	1.09 (1.05, 1.13)
64–74 mmol/l (8.0–8.9%)	0.98 (0.93, 1.03)	1.12 (1.08, 1.16)
≥75 mmol/l (≥9.0%)	1.15 (1.09, 1.20)	1.17 (1.13, 1.22)
BMI		
Normal weight	Reference	Reference
Overweight	0.72 (0.69, 0.76)	0.92 (0.89, 0.95)
Obese class I	0.65 (0.61, 0.68)	0.88 (0.85, 0.91)
Obese class II/III	0.64 (0.61, 0.67)	0.91 (0.88, 0.94)
eGFR (ml/min per 1.73 m^2^)		
≥90	Reference	Reference
60–89	0.95 (0.92, 0.98)	0.99 (0.97, 1.02)
30–59	0.95 (0.91, 1.00)	1.06 (1.02, 1.09)
15–29	1.08 (0.95, 1.22)	1.14 (0.95, 1.38)
<15	1.58 (1.05, 2.37)	0.46 (0.12, 1.78)
Smoking	1.20 (1.16, 1.24)	1.15 (1.12, 1.18)

Values are HR (95% CI). Detailed definitions of characteristics are provided in ESM Table 2

^a^The Nordic countries are Sweden, Norway, Denmark and Finland

^b^Platelet inhibitors, statins, ACE inhibitors/angiotensin II receptor blockers, calcium antagonists, β-blockers

^c^Not including insulin in both cohorts, SGLT2 inhibitors in the SGLT2 inhibitor cohort, and GLP-1 receptor agonists in the GLP-1 receptor agonist cohort

ACE, angiotensin-converting-enzyme; NDR, National Diabetes Register

ESM Figs 1 and 2 shows that calendar year of drug initiation had little effect on the cumulative incidence of treatment discontinuation for both study drugs.

Table 2 shows that several variables were associated with a higher or lower risk for treatment discontinuation, with the associations being of similar direction and magnitude for both GLP-1 receptor agonist users and SGLT2 inhibitors users. Examples of variables associated with a higher or lower risk for treatment discontinuation for both drugs were age, place of birth, education, income and number of diabetes drugs used.

### Treatment reinitiation

Among those who discontinued a GLP-1 receptor agonist, the cumulative incidence of reinitiating treatment was 41.1% (95% CI 40.5, 41.7) at 1 year and 57.4% (95% CI 56.7, 58.1) at 3 years when using a grace period of 90 days in the definition of treatment discontinuation (Fig. 1 and ESM Table 5). Similarly, among those who discontinued an SGLT2 inhibitor, the cumulative incidence of reinitiating treatment was 40.4% (95% CI 39.9, 40.8) at 1 year and 55.7% (95% CI 55.2, 56.2) at 3 years when using a grace period of 90 days (Fig. 2 and ESM Table 5). In line with the analyses of treatment discontinuation, the proportion of patients reinitiating treatment were substantially reduced or increased, respectively, when applying longer and shorter grace periods. When using a grace period of 60, 90 or 180 days to define treatment discontinuation, most treatment reinitiations occurred within the first year after discontinuation, but this pattern was not seen with a grace period of 365 days.

### Proportion of patients covered

Figures 1 and 2 show the proportion of patients who were covered among GLP-1 receptor agonist users and SGLT2 inhibitor users, respectively. For GLP-1 receptor agonists, the proportion of patients covered was 81.7% at 1 year, 76.7% at 3 years and 75.1% at 5 years when using a grace period of 90 days. For SGLT2 inhibitors, the proportion of patients covered was 78.5% at 1 year, 69.8% at 3 years and 68.2% at 5 years when using a grace period of 90 days. The results were largely similar across the various lengths of grace period for both drugs (ESM Table 6).

### Adherence

Among 72,733 GLP-receptor agonist users who were alive 1 year after the date of filling their first prescription, 68% had ≥80% of proportion of days covered, 60% had ≥90% proportion of days covered, and 22% had 100% proportion of days covered. The 1-year mean proportion of days covered was 78% (SD 39) and the 1-year median proportion of days covered was 95% (IQR 63–100).

Among 111,104 SGLT2 inhibitor users who were alive 1 year after the date of filling their first prescription, 64% had ≥80% of proportion of days covered, 58% had ≥90% proportion of days covered, and 33% had 100% proportion of days covered. The 1-year mean proportion of days covered was 77% (SD 30) and the 1-year median proportion of days covered was 97% (IQR 55–100).

ESM Table 7 shows the mean and median proportions of days covered across subgroups of ASCVD status, chronic kidney disease status, heart failure status, BMI category and year of initiation.

### Treatment trajectories and switching of drug within the same drug class

Between 1 January 2017 and 31 December 2019, 35,105 patients initiated treatment with a GLP-1 receptor agonist (liraglutide: 22,008 [62.7%]; semaglutide: 6714 [19.1%]; other GLP-1 receptor agonist: 6383 [18.2%]) and 55,988 patients initiated treatment with an SGLT2 inhibitor (empagliflozin: 48,499 [86.6%]; dapagliflozin: 6929 [12.4%]; other SGLT2 inhibitors: 560 [1.0%]).

**Fig. 5:**
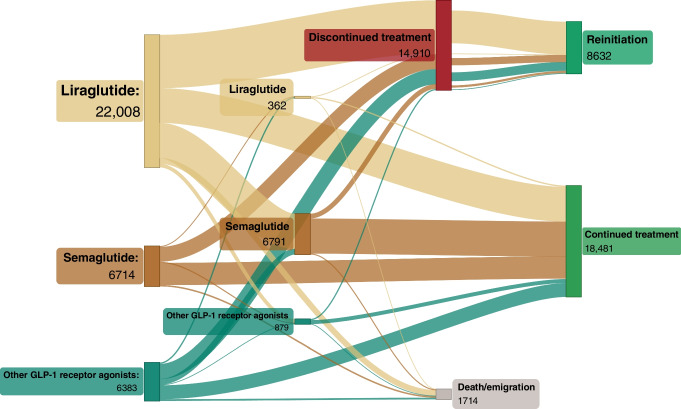
Treatment trajectories and switching between drugs within the same drug class for GLP-1 receptor agonists. ‘Other GLP-1 receptor agonists’ comprise exenatide, lixisenatide and dulaglutide

At 3 years after drug initiation, of the 35,105 GLP-1 receptor agonist users, 14,910 (42.5%) had discontinued treatment, 18,481 (52.6%) had continued treatment and 1714 (4.9%) had died or emigrated (Fig. 5). A total of 8032 (22.9%) patients had switched GLP-1 receptor agonist, with the most common drug switch being from liraglutide to semaglutide (*n*= 5839; 26.5% of all liraglutide initiators). Of those who had discontinued treatment, 8632 (57.9%) later reinitiated treatment but 6278 (42.1%) did not.

**Fig. 6:**
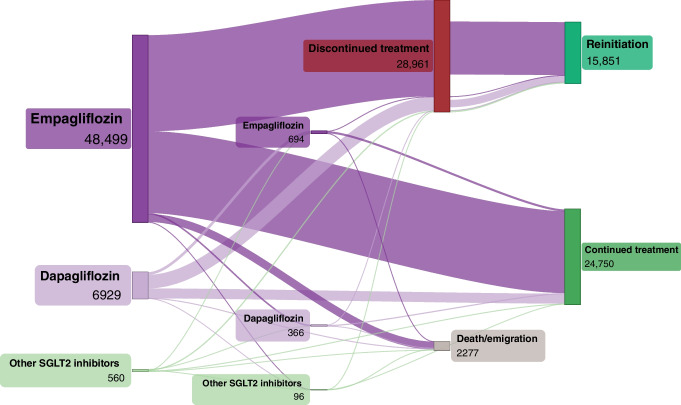
Treatment trajectories and switching between drugs within the same drug class for SGLT2 inhibitors. ‘Other SGLT2 inhibitors’ comprise canagliflozin and ertugliflozin

At 3 years after drug initiation, of the 55,988 SGLT2 inhibitor users, 28,961 (51.7%) had discontinued treatment, 24,750 (44.2%) had continued treatment and 2277 (4.1%) had died or emigrated (Fig. 6). A total of 1156 (2.1%) patients had switched to another SGLT2 inhibitor. Of those who had discontinued treatment, 15,851 (54.7%) later reinitiated treatment but 13,110 (45.3%) did not.

## Discussion

This nationwide study, which includes patients with type 2 diabetes who initiated treatment with GLP-1 receptor agonists or SGLT2 inhibitors, had several key findings. First, approximately 1 in 4 patients had discontinued treatment at 1 year after treatment initiation, and almost half had discontinued at 3 years, with these findings being largely similar for GLP-1 receptor agonists and SGLT2 inhibitors. Approximately half of those who had discontinued treatment had reinitiated treatment within 3 years after discontinuation, such that the proportion of patients who had ongoing treatment (proportion of patients covered) was approximately 70–80% for both drugs during a 1–5 year period after treatment initiation. Second, the length of the grace period, i.e. the time allowed between and after prescriptions when defining ongoing treatment, had a substantial impact on the observed rates of both treatment discontinuation and reinitiation. Third, both the rates of, and factors associated with, treatment discontinuation were similar for GLP-1 receptor agonists and SGLT2 inhibitors, indicating that patient-specific factors, rather than drug-specific factors (such as side-effects), are driving treatment discontinuation. The exception was higher BMI, which showed a stronger association with lower discontinuation rates among GLP-1 receptor agonist users than SGLT2 inhibitor users. Finally, for GLP-1 receptor agonists, switching between drugs within the same drug class was common, with approximately 1 in 4 patients who initiated treatment with a GLP-1 receptor agonist in our study having switched drug at least once.

The technical definition of treatment discontinuation had a substantial impact on the findings in our study. Varying the grace period between 60 and 365 days meant that the discontinuation rates at 3 years after treatment initiation ranged from 23% to 44% among GLP-1 receptor agonist users and from 29% to 51% among SGLT2 inhibitor users. However, varying the length of grace period had a less pronounced effect on the findings when assessing the proportion of patients with ongoing treatment (proportion of patients covered [31]). Moreover, there were consistently larger proportions of patients covered by treatment than there were patients who had not discontinued treatment (estimated as 1 minus the cumulative incidence of treatment discontinuation) for both study drugs, with these differences increasing over the follow-up period. This was because the proportion of patients covered also accounts for those who have reinitiated treatment after having discontinued it, thus including all patients with ongoing treatment at a given time point after treatment initiation. These findings indicate that studies using routine clinical practice data should present analyses for a range of grace periods, and provide drug utilisation estimates that account for both treatment discontinuation and reinitiation and are less sensitive to the technical definition of treatment discontinuation [31], rather than focusing on one arbitrary definition of treatment discontinuation. Our findings also indicate that there is no obvious difference between treatment discontinuation and low adherence in this type of data: whether patients who have long gaps between filled prescriptions should be considered as having discontinued and reinitiated treatment or as having poor adherence is not clear.

Against this background, comparisons of findings between our and previous studies, which have used various grace periods [32–36], are not straightforward. Two studies from Denmark [32, 36], using a grace period of 90 days (as was done in our main analyses), reported that approximately 20% of both GLP-1 receptor agonist users [32, 36] and SGLT2 inhibitor users [32] had discontinued treatment within 1 year of treatment initiation. In another study using data from Stockholm, Sweden, a shorter grace period of 60 days was used, and the 1-year discontinuation rates were higher (34% for GLP-1 receptor agonist users and 35% for SGLT2 inhibitor users) [33].

Compared with clinical trials [7–18], we observed a higher rate of treatment discontinuation for both GLP-1 receptor agonists and SGLT2 inhibitors. The lower rate of treatment discontinuation in the trials may potentially be explained by patient monitoring and encouragement to adhere to the protocol, and/or selective inclusion of patients who are more likely to adhere to treatment.

GLP-1 receptor agonists and SGLT2 inhibitors have different mechanisms of action and side-effect profiles. Treatment discontinuation for GLP-1 receptor agonists was lower among patients with high BMI, while associations of similar magnitude were not observed for SGLT2 inhibitors. This finding may reflect the possibility that the perceptible benefit of weight loss associated with GLP-1 receptor agonist use motivates continued treatment. In contrast, for most other patient characteristics assessed in our study, the associations with treatment discontinuation were similar for SGLT2 inhibitors and GLP-1 receptor agonists. Moreover, the overall discontinuation rates were similar for the two drugs. Although the occurrence of different side-effects can lead to similar overall discontinuation rates, these findings raise the possibility that a large part of the treatment discontinuation is driven by patient-specific factors that affect treatment adherence across various types of drugs rather than drug-specific factors (including side-effects or oral vs subcutaneous administration). Examples of patient-specific factors that potentially affect treatment discontinuation and adherence are the inability or reluctance to cover drug costs, polypharmacy, changes in medical needs, language barriers leading to miscommunication with health professionals, and patient priorities. Importantly, it should be noted many cases of treatment discontinuation are related to the patient’s medical needs and may not be due to low patient adherence to the prescribed treatment.

The most common switch within the same drug class was seen among GLP-1 receptor agonists, specifically from liraglutide to semaglutide. This may potentially be attributed to physicians recommending semaglutide due to its greater effects on HbA_1c_ levels and body weight. Additionally, patients may prefer the weekly injection of semaglutide over the daily injections of liraglutide, as indicated by the lower discontinuation rates for weekly vs daily injections of GLP-1 receptor agonists observed in some studies [36–39].

Future studies may be necessary to outline and assess interventions aimed at reducing discontinuation and improving adherence. To develop more personalised interventions, analytical methods such as group-based trajectory modelling could be used to group patients based on discontinuation and adherence patterns over time [40].

### Strengths and limitations

Strengths of this study are the use of a large, unselected study population from routine clinical practice that comprises almost all patients with type 2 diabetes in Sweden, and the use of nationwide registers that provide high-quality data on prescription drug use, diagnoses registered during hospitalisation and outpatient visits, and clinical variables such as BMI and HbA_1c_, as well as markers of socioeconomic status.

This study was not without limitations. While we had complete coverage of data on filled prescriptions, information about if and how the patients took the drugs was not available. We also had no information on reasons for treatment discontinuation. Further, although diagnoses and procedure codes registered in the Swedish Patient Register have high sensitivity and positive predictive values [25], our findings may be affected by misclassification of certain patient comorbidities that were defined using these registers. Finally, Sweden provides universal healthcare with very low patient costs for healthcare visits and prescription drugs; it may therefore be hypothesised that there are higher discontinuation rates and lower reinitiation rates in some other countries.

### Conclusions

Approximately half of type 2 diabetes patients who had started using GLP-1 receptor agonists or SGLT2 inhibitors had discontinued treatment within 5 years of follow-up. However, more than half of those who discontinued subsequently reinitiated treatment, such that the proportion with ongoing treatment was approximately 70–80% for both drugs during a 1–5 year period after treatment initiation. This suggests that the proportion of patients with long-term use of the medications is larger than indicated by analyses that focus on treatment discontinuation. Patient characteristics associated with treatment discontinuation were similar for GLP-1 receptor agonists and SGLT2 inhibitors.

## Supplementary Information

Below is the link to the electronic supplementary material.

Supplementary file1 (PDF 545 KB)

## Data Availability

The data analysed in this study are based on Swedish nationwide registers. Individual-level data in the registers can only be accessed through secure servers, and only export of aggregated data, as presented in research articles, is allowed according to Swedish law. Permission to access data can be made only after fulfilling specific requirements to safeguard the anonymity of the study participants. For these reasons, data cannot be made generally available.

## Abbreviations

ASCVD: Atherosclerotic cardiovascular disease
GLP-1: Glucagon-like peptide-1
SGLT2: Sodium-glucose cotransporter 2
