# Supplementary material for: Treatment discontinuation among users of GLP-1 receptor agonists and SGLT2 inhibitors in a national population of individuals with type 2 diabetes
Source: Diabetologia. 2025 May 2;68(8):1680–95. doi: 10.1007/s00125-025-06439-x (PMC12246030; doi:10.1007/s00125-025-06439-x)

ELECTRONIC SUPPLEMENTARY MATERIAL

Lim et al. Treatment discontinuation among users of GLP-1 receptor agonists and SGLT2 inhibitors in a national population of patients with type 2 diabetes.

Table of Contents

ESM Table 1 ATC codes for study drugs and estimated days of supply 3

ESM Table 2 Definition of variables used for description of population characteristics and Fine-Gray subdistribution hazard model with proportions missing 4

ESM Table 3 Definitions for atherosclerotic cardiovascular disease (ASCVD), chronic kidney disease, and heart failure. (10-year look-back for ICD-10 and procedure codes in the Patient Register; 365 days for variables in the National Diabetes Register) 7

ESM Table 4 Cumulative incidence of treatment discontinuation across various lengths of the grace period used to define treatment discontinuation among new users of GLP-1 receptor agonists and SGLT2 inhibitors 8

ESM Table 6 Proportion of patients covered for GLP-1 receptor agonist and SGLT2 inhibitor users 10

ESM Table 7 Proportion of days covered at 1 year after the date of filling the first prescription of a GLP-1 receptor agonist or an SGLT2 inhibitor 11

ESM Fig. 1 Cumulative incidence of treatment discontinuation across year of initiation among GLP-1 receptor agonist users 12

ESM Fig. 2 Cumulative incidence of treatment discontinuation across year of initiation among SGLT2 inhibitor users 13

ESM Table 1 ATC codes for study drugs and estimated days of supply

	ATC code	Days of supply
**GLP-1 receptor agonists**	Exenatide	
	A10BJ01	0.5 per dose if 5 or 10 μg and 7.0 per dose if 2 mg
	Liraglutide	
	A10BJ02 (Victoza)	5.0 per ml
	A10BJ02 (Saxenda)	2.0 per ml
	A10AE56	3.0 per ml
	Lixisenatide	
	A10BJ03	1.0 per dose
	A10AE54	5.0 per 3 ml
	Dulaglutide	
	A10BJ05	7.0 per dose
	Semaglutide	
	A10BJ06 (injectable)	7.0 per dose
	A10BJ06 (oral)	1.0 per tablet
**SGLT2 inhibitors**	Dapagliflozin	
	A10BK01	1.0 per tablet
	A10BD15	0.5 per tablet
	A10BD21	1.0 per tablet
	Canagliflozin	
	A10BK02	1.0 per tablet
	A10BD16	0.5 per tablet
	Empagliflozin	
	A10BK03	1.0 per tablet
	A10BD19	1.0 per tablet
	A10BD20	0.5 per tablet
	Ertugliflozin	
	A10BK04	1.0 per tablet
	A10BD23	0.5 per tablet

ESM Table 2 Definition of variables used for description of population characteristics and Fine-Gray subdistribution hazard model with proportions missing

**Sociodemographic characteristics**	**ICD/categories**	**Missing in the GLP-1 receptor agonist cohort, %**	**Missing in the SGLT2 inhibitor cohort, %**
Sex	Women; men	0	0
Age (years)	40-49, 50-59, 60-69, 70-79, ≥80	0	0
Place of birth	Nordic countries (Sweden, Norway, Denmark, Finland); Rest of Europe; Outside Europe	0.05	0.05
Education	Primary school and high school; vocational or short-term tertiary education; medium or long-term tertiary education	1.6	1.9
Income	Income levels were categorized into quartiles within both sex and each age group as specified above. For example, if a male patient is 45 years old, the patient is categorized to high income if they are in the 4th quartile of income among males ages 40-49.<br>High – 4th quartile<br>Medium – 2nd and 3rd quartile<br>Low – 1st quartile	0.2	0.2
Living with partner	Yes; no	0.2	0.2
**Medical history (10-year look-back)**	*ICD-10 code and procedure code*		
Ischaemic heart disease and coronary revascularization	ICD-10: I20-I25; procedure FNA, FNB, FNC, FND, FNE, FNG, FNP02, FNP12, FNQ05, FNQ12, FNR22	0	0
Ischaemic stroke	ICD-10: I63, I693	0	0
Arterial disease (including amputation)	ICD-10: I65, I70, I72, I73, I74, I77, K550, K551, E115, E135, E145; procedure code: NFQ, NGQ, NHQ	0	0
Atrial fibrillation	ICD-10: I48	0	0
Heart failure	ICD-10: I110, I130, I132, I50	0	0
Chronic kidney disease	eGFR <60 ml/min/1.73 m^2 or macroalbuminuria or ICD-10: Z49, Z940, Z992; procedure code: KAS, DR012, DR013, DR014, DR015, DR016, DR023, DR024, DR055, DR056, DR060, DR061	28.6	30.0
Liver disease	ICD-10: B15-B19, K70-K77, C22	0	0

Pancreatitis	ICD-10: K85	0	0
Diabetic eye complications	ICD-10: E113, E133, E143, H280, H358, H360<br><br>Procedure code: CKC12, CKD65	0	0
Other diabetic complications	ICD-10: E110 (excl E110A), E111 (excl E111A), E130, E131, E140, E141, E160, E161, E162, E116, E117, E118, G990, G590, G632, E114, E134, E136, E137, E138, E144, E146, E147, E148, M142, M146, M908, L984	0	0
Psychiatric disorder	ICD-10: F00-F09, F20- F99	0	0
Mental and behavioral disorders due to psychoactive substance use	ICD-10: F10-F19	0	0
**Medications in the last 12 months**	*ATC code*		
SGLT2 inhibitor[a]	Dapagliflozin: A10BK01, A10BD15, A10BD21, *A10BD25*; Canagliflozin: A10BK02, A10BD16; Empagliflozin: A10BK03, A10BD19, A10BD20, *A10BD27*; Ertugliflozin: A10BK04, A10BD23, A10BD24; Ipragliflozin: *A10BK05*; Sotagliflozin: A10BK06; Luseogliflozin: *A10BK07*	0	0
GLP-1 receptor agonist[a]	Exenatide: A10BJ01; Liraglutide: A10BJ02, A10AE56; Lixisenatide: A10BJ03, A10AE54; *Albiglutide: A10BJ04;* Dulaglutide: A10BJ05; Semaglutide: A10BJ06; *Beinaglutide: A10BJ07*	0	0
Insulin[a]	*A10AA*, A10AB, A10AC, A10AD, A10AE, *A10AF*	*0*	*0*
Other non-insulin diabetes drugs (metformin, sulfonylureas, DPP4 inhibitors, glitazones, glinides, acarbose)	Glitazones, glinides, acarbose: A10BF, A10BG, *A10BD03*, *A10BD04*, A10BD05, A10BD06, A10BD09, *A10BD12, A10BD14*, *A10BD17*, *A10BD26*, A10BX<br><br>DPP4 inhibitors: A10BH, A10BD07, A10BD08, A10BD09, A10BD10, A10BD11, *A10BD12*, A10BD13, *A10BD18*, A10BD19, A10BD21, *A10BD22*, A10BD24, *A10BD25*, *A10BD27*<br><br>Metformin: A10BA02, *A10BD02*, *A10BD03*, A10BD05, A10BD07, A10BD08, A10BD10, A10BD11, A10BD13, *A10BD14*, A10BD15, A10BD16, *A10BD17*, *A10BD18*, A10BD20, *A10BD22*, A10BD23, *A10BD25*, *A10BD26*, *A10BD27*<br><br>Sulfonylureas: A10BB, A10BD01, A10BD02, A10BD04, A10BD06	0	0

Time since first diabetes drug	A10;<br><1 year, ≥1 to <10 years, ≥10 years	0	0
Number of diabetes drugs[b]	0; 1 or 2, ≥3	0	0
Platelet inhibitors[c]	B01AC24, B01AC04, B01AC22	0	0
Statins[c]	C10AA, C10B	0	0
ACE inhibitors/Angiotensin II receptor blockers[c]	C09A, C09B, C09C, C09D	0	0
Calcium antagonists[c]	C08C, C08D, C09BB, C09DB, C09DX01	0	0
β-blockers[c]	C07	0	0
Diuretics	C03C, C03EB, C03A, C03B, C03D, C03EA	0	0
**National Diabetes Register variables (1-year look back)**			
Blood pressure	Normotension: SBP <140 mmHg AND DBP <90 mmHg<br>Stage 1 hypertension: SBP ≥140 to <160 mmHg or DBP: ≥90 to <100mmHg<br>Stage 2 hypertension: SBP ≥160 mmHg or DBP: ≥100 mmHg	14.7	15.3
HbA1c	≤48 mmol/mol (≤6.5%)<br>49-52 mmol/mol (6.6-6.9%)<br>53-63 mmol/mol (7.0-7.9%)<br>64-74 mmol/mol (8.0 – 8.9%)<br>≥75 mmol/mol (≥9.0%)	11.2	12.4
Body mass index	Normal weight: <25 kg/m^2<br>Overweight: ≥25 to <30 kg/m^2<br>Obese class I: ≥30 to <35 kg/m^2<br>Obese class II/III: ≥35 kg/m^2	20.3	21.9
eGFR (ml/min per $1.73m^2$)	≥90; 60 to <90; 30 to <60; <30	16.5	17.3
Smoking	Yes/No	21.7	22.1

Abbreviations: DDP4, dipeptidyl peptidase 4; eGFR, estimated glomerular filtration rate; GLP-1, glucagon-like peptide; HbA1c, glycated haemoglobin; SGLT2 sodium-glucose co-transporter 2.

[a]Not assessed for in Fine-Gray subdistributional hazard model
[b]Not including insulin in both cohorts and SGLT2 inhibitors in the SGLT2 inhibitor cohort and not GLP-1 receptor agonists in the GLP-1 receptor agonist cohort
[c]Merged into single-variable "Cardiovascular drugs" for in Fine-Gray subdistributional hazard model

ESM Table 3 Definitions for atherosclerotic cardiovascular disease (ASCVD), chronic kidney disease, and heart failure. (10-year look-back for ICD-10 and procedure codes in the Patient Register; 365 days for variables in the National Diabetes Register)

	**ICD 10/procedure code**
Atherosclerotic cardiovascular disease (ASCVD)	Coronary revascularization: procedure code: FNA, FNB, FNC, FND, FNE, FNG, FNP02, FNP12, FNQ05, FNQ12, FNR22<br>Ischaemic heart disease: I20-I25<br>Ischaemic stroke: I63, I69.3<br>Arterial disease, including amputation: I65, I70, I72, I73, I74, I77, K550, K551, E115, E135, E145; procedure code: NFQ, NGQ, NHQ
Chronic kidney disease	eGFR <60 ml/min/1.73 m^2 or macroalbuminuria or ICD-10: Z49, Z940, Z992; procedure code: KAS, DR012, DR013, DR014, DR015, DR016, DR023, DR024, DR055, DR056, DR060, DR061
Heart failure	I110, I130, I132, I50

ESM Table 4 Cumulative incidence of treatment discontinuation across various lengths of the grace period used to define treatment discontinuation among new users of GLP-1 receptor agonists and SGLT2 inhibitors

**Number of years after filling of first prescription**	**GLP-1 receptor agonists**	**SGLT2 inhibitors**
	Cumulative incidence % (95% CI)	Cumulative incidence % (95% CI)
Grace period of 60 days		
1 year	28.1 (27.7-28.4)	33.7 (33.4-34.0)
2 years	38.0 (37.7-38.4)	44.8 (44.5-45.1)
3 years	43.6 (43.2-44.0)	50.6 (50.3-50.9)
4 years	47.5 (47.0-47.9)	54.8 (54.5-55.2)
5 years	50.3 (49.8-50.8)	57.8 (57.4-58.2)
Grace period of 90 days		
1 year	23.6 (23.2-23.9)	27.9 (27.6-28.1)
2 years	33.2 (32.8-33.5)	39.7 (39.4-40.0)
3 years	38.5 (38.2-38.9)	45.9 (45.5-46.2)
4 years	42.3 (41.9-42.7)	50.2 (49.8-50.5)
5 years	45.3 (44.8-45.8)	53.3 (52.9-53.7)
Grace period of 180 days		
1 year	16.2 (16.0-16.5)	18.8 (18.6-19.0)
2 years	26.0 (25.6-26.3)	30.7 (30.5-31.0)
3 years	31.0 (30.6-31.4)	36.8 (36.5-37.1)
4 years	34.8 (34.4-35.2)	41.0 (40.7-41.4)
5 years	37.8 (37.3-38.3)	44.2 (43.8-44.5)
Grace period of 365 days		
1 year	-	-
2 years	17.8 (17.5-18.1)	21.5 (21.3-21.8)
3 years	23.3 (23.0-23.7)	28.8 (28.5-29.1)
4 years	26.9 (26.5-27.3)	33.1 (32.8-33.4)
5 years	29.8 (29.3-30.3)	36.2 (35.8-36.6)

ESM Table 5 Cumulative incidence of treatment re-initiation across various lengths of the grace period used to define treatment discontinuation among those who discontinued treatment with an GLP-1 receptor agonist and an SGLT2 inhibitor

**Number of years after discontinuing treatment**	**GLP-1 receptor agonists**	**SGLT2 inhibitors**
	Cumulative incidence % (95% CI)	Cumulative incidence % (95% CI)
Grace period of 60 days		
1 year	49.0 (48.4-49.6)	47.3 (46.9-47.8)
2 years	57.2 (56.6-57.8)	55.1 (54.7-55.5)
3 years	63.0 (62.4-63.7)	60.9 (60.4-61.4)
4 years	67.5 (66.8-68.2)	65.7 (65.1-66.2)
5 years	70.7 (69.8-71.6)	69.8 (69.1-70.5)
Grace period of 90 days		
1 year	41.1 (40.5-41.7)	40.4 (39.9-40.8)
2 years	50.6 (50.0-51.3)	49.1 (48.6-49.5)
3 years	57.4 (56.7-58.1)	55.7 (55.2-56.2)
4 years	62.4 (61.6-63.2)	61.1 (60.5-61.7)
5 years	66.4 (65.3-67.5)	66.1 (65.3-66.9)
Grace period of 180 days		
1 year	28.2 (27.5-28.8)	26.9 (26.4-27.4)
2 years	39.7 (38.9-40.4)	37.2 (36.7-37.7)
3 years	48.5 (47.6-49.4)	45.6 (45.0-46.2)
4 years	53.7 (52.7-54.7)	52.3 (51.6-53.1)
5 years	58.9 (57.4-60.4)	57.9 (56.8-59.0)
Grace period of 365 days		
1 year	18.6 (17.9-19.3)	17.3 (16.9-17.8)
2 years	31.2 (30.3-32.1)	28.7 (28.1-29.3)
3 years	40.3 (39.2-41.4)	38.2 (37.4-39.0)
4 years	46.8 (45.4-48.3)	46.1 (45.0-47.2)
5 years	52.9 (49.8-55.9)	51.7 (49.4-54.0)

ESM Table 6 Proportion of patients covered for GLP-1 receptor agonist and SGLT2 inhibitor users

**Number of years after filling of first prescription**	**GLP-1 receptor agonists**	**SGLT2 inhibitors**
	Proportion of patients covered, %	Proportion of patients covered, %
Grace period of 60 days		
1 year	80.3	76.6
2 years	76.8	70.9
3 years	75.7	68.7
4 years	75.3	67.3
5 years	74.0	67.2
Grace period of 90 days		
1 year	81.7	78.5
2 years	77.9	72.2
3 years	76.7	69.8
4 years	76.3	68.4
5 years	75.1	68.2
Grace period of 180 days		
1 year	85.5	83.0
2 years	80.1	74.9
3 years	78.8	72.1
4 years	78.3	70.7
5 years	76.8	70.2
Grace period of 365 days		
1 year	100.0	100.0
2 years	84.2	80.0
3 years	81.8	75.5
4 years	81.0	73.6
5 years	79.6	72.6

ESM Table 7 Proportion of days covered at 1 year after the date of filling the first prescription of a GLP-1 receptor agonist or an SGLT2 inhibitor

	**GLP-1 receptor agonists, *N* = 72,733**		**SGLT2 inhibitors, *N* = 111,104**	
	Mean (SD)	Median (IQR)	Mean (SD)	Median (IQR)
**Overall**	0.78 (0.30)	0.95 (0.63, 1.00)	0.77 (0.30)	0.97 (0.55, 1.00)
ASCVD status				
ASCVD	0.77 (0.31)	0.95 (0.60, 1.00)	0.79 (0.30)	0.98 (0.58, 1.00)
No ASCVD	0.79 (0.29)	0.95 (0.66, 1.00)	0.77 (0.30)	0.96 (0.54, 1.00)
Chronic kidney disease status				
Chronic kidney disease	0.78 (0.31)	0.96 (0.62, 1.00)	0.77 (0.31)	0.97 (0.53, 1.00)
No chronic kidney disease	0.79 (0.29)	0.95 (0.64, 1.00)	0.78 (0.30)	0.97 (0.56, 1.00)
Heart failure status				
Heart failure	0.77 (0.30)	0.95 (0.61, 1.00)	0.81 (0.29)	0.99 (0.65, 1.00)
No heart failure	0.78 (0.30)	0.95 (0.64, 1.00)	0.77 (0.30)	0.96 (0.54, 1.00)
Body mass index (kg/m^2)				
Normal weight	0.71 (0.34)	0.90 (0.38, 0.99)	0.75 (0.31)	0.96 (0.49, 1.00)
Overweight	0.77 (0.31)	0.95 (0.60, 1.00)	0.77 (0.30)	0.97 (0.54, 1.00)
Obese class I	0.79 (0.29)	0.96 (0.68, 1.00)	0.78 (0.29)	0.97 (0.58, 1.00)
Obese class II/III	0.80 (0.28)	0.96 (0.69, 1.00)	0.77 (0.30)	0.96 (0.56, 1.00)
Year of initiation				
2017	0.78 (0.30)	0.94 (0.62, 0.99)	0.76 (0.31)	0.96 (0.54, 1.00)
2018	0.79 (0.29)	0.95 (0.64, 1.00)	0.77 (0.30)	0.96 (0.56, 1.00)
2019	0.79 (0.30)	0.95 (0.63, 1.00)	0.77 (0.30)	0.97 (0.54, 1.00)
2020	0.79 (0.30)	0.96 (0.66, 1.00)	0.77 (0.30)	0.96 (0.54, 1.00)
2021	0.78 (0.30)	0.96 (0.62, 1.00)	0.78 (0.29)	0.98 (0.58, 1.00)

ESM Fig. 1 Cumulative incidence of treatment discontinuation across year of initiation among GLP-1 receptor agonist users

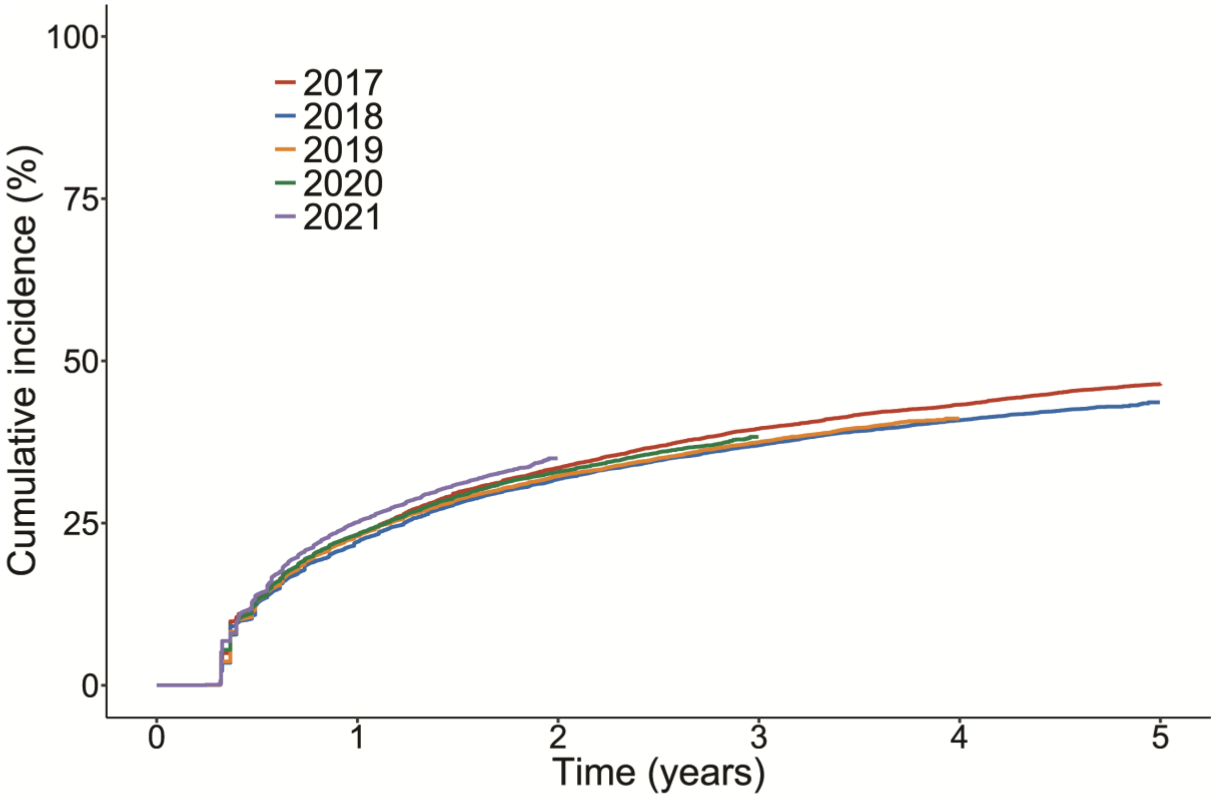

Number at risk

2017	8885	6739	5743	5123	4713	4341
2018	11260	8636	7406	6679	6120	0
2019	14960	11319	9743	8797	0	0
2020	17026	12814	11016	0	0	0
2021	21764	16017	0	0	0	0

ESM Fig. 2 Cumulative incidence of treatment discontinuation across year of initiation among SGLT2 inhibitor users

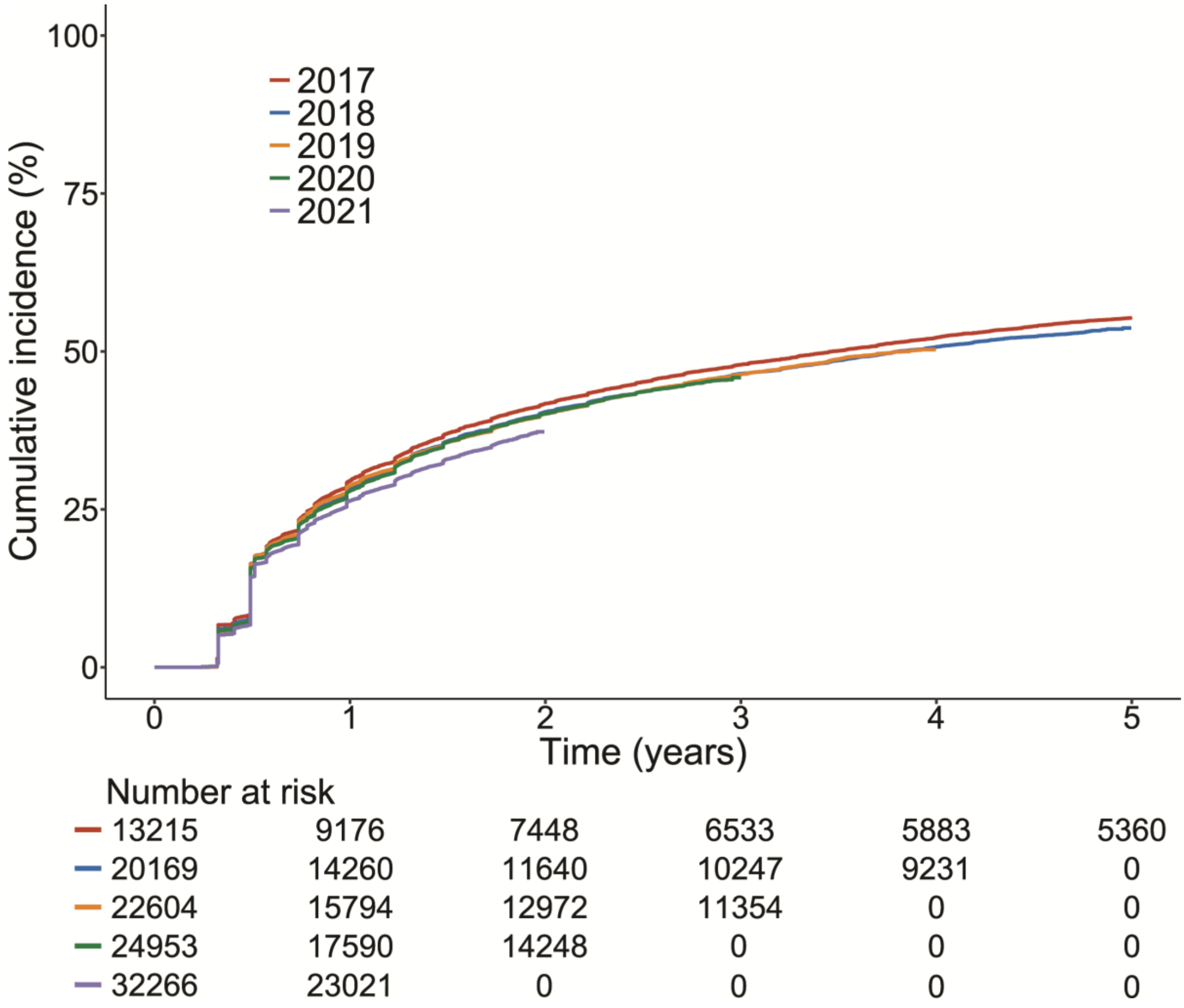

Supplement: Supplementary file 1 — Supplementary file1 (PDF 545 KB) [file 125_2025_6439_MOESM1_ESM.pdf]
